# Alcohol Dehydrogenase Activity and Electrical Conductivity of Seed Exudates as Potential Indicators of Flooding Tolerance in Soybean Groups During the Germination Stage

**DOI:** 10.3390/plants15213302

**Published:** 2026-10-26

**Authors:** Weeraphorn Jira-anunkul, Khundej Suriharn, Wattana Pattanagul, Sompong Chankaew

**Affiliations:** 1 Department of Agronomy, Faculty of Agriculture, Khon Kaen University, Khon Kaen 40002, Thailand; weeraji@kku.ac.th (W.J.-a.); sphala@kku.ac.th (K.S.); 2 Plant Breeding Research Center for Sustainable Agriculture, Khon Kaen University, Khon Kaen 40002, Thailand; 3 Department of Biology, Faculty of Science, Khon Kaen University, Khon Kaen 40002, Thailand; w@kku.io

**Keywords:** aerobic respiration, alcohol dehydrogenase, electrical conductivity, flooding screening, interval flooding, *Glycine max*

## Abstract

Flooding stress is one of the abiotic stresses that limit soybean growth and development, particularly in seed germination. Excess water in soil depletes oxygen, leading to germination failure and uneven plant populations and establishment. Previous studies have evaluated the effects of flooding stress on germination in the field and across multiple growth stages. However, the response of soybean germination under flooding stress between tolerance levels is not well understood. In this study, seed soaking was performed in the laboratory to simulate flooding stress. Fifteen soybean varieties, grouped by seed soaking tolerance (tolerant, moderate, and susceptible), were investigated. Each soybean group was exposed to seed soaking for 12, 24, 36, and 48 h. Imbibition curve, electrical conductivity (EC), alcohol dehydrogenase activity (ADH), and ethanol content were analyzed. The results showed that ADH activity in cotyledon tissues and EC differed between groups. ADH in cotyledon tissues of the moderate and susceptible groups was induced at 12 h, whereas under stress, it was induced at 48 h in the tolerant group. Interval EC measurements from seed exudates indicated that tolerant varieties had lower conductivity than the moderate and susceptible groups. In summary, ADH activity in cotyledon tissues at 48 h and EC can serve as indirect physiological traits for discriminating flooding-tolerant groups during germination. KKU–SB052 is recommended as a natural genetic resource for soybean breeding programs, as indicated by high ADH activity after prolonged flooding and maintenance of cell membrane integrity under flooding stress.

## 1. Introduction

Soybeans (*Glycine max* (L.) Merr.) are generally susceptible to flooding stress. Climate change is increasing the frequency of heavy precipitation, with predictions indicating a 30% rise in heavy rainfall by 2030 [1]. Flooding reduces soybean productivity by approximately 16%, causing billion-dollar losses for farmers [2]. Soybean production is dominated in temperate regions due to cool, moderate climates. However, production is also increasing in tropical areas to meet the growing demand in developing regions [3]. Climate change affects soybean production through temperature fluctuations, altered precipitation patterns, and extreme weather events such as droughts, floods, storms, and hail [4]. Flooding significantly impacts soybean growth and yield. For instance, soybeans are cultivated in flood-prone areas in the Huanghua Hai Plain in China and Indonesia [5,6,7]. Similarly, the soybean cultivation system in Thailand is adopted in rice–soybean crop rotation or planting in flooded irrigated rice areas. The main challenge in this crop rotation system is poor natural water drainage and exposure to temporary flooding. According to our observations of farmer practices, farmers flood the rice paddy with water, maintaining a depth of approximately 2–3 cm from the soil surface, and they leave it until the soil is fully saturated. Then, soybean seeds are sown in the flooded paddy field and left for 2–3 h or more before the water is drained. This results in a lower germination rate in this cultivated system and requires using higher seed rates to maintain seedling populations. Previous studies reported that soybean seeds subjected to 1 day (24 h) of flooding showed a 20% reduction in germination. Furthermore, the germination rate continued to decline to 50–70% after 2–3 days of flooding stress. SJ5 and CM60 (Thailand’s commercial soybean varieties) had germination percentages of 79.5% and 67.0%, respectively, after 2 h of soaking in water. In addition, no germination was observed when soybeans were planted at 100% soil water–holding capacity [8,9].

Flooding reduces oxygen availability (hypoxia), and prolonged flooding in the soil may lead to the absence of oxygen (anoxia). Oxygen limitation impairs gas exchange between the atmosphere and the seed coat, reducing ATP synthesis. The energy crisis suffered by seeds is due to oxygen deprivation [1,10]. The fermentation pathways not only occur under normal oxygen levels but are also rapidly induced in response to low oxygen levels to supply energy for survival under limited oxygen conditions [11]. Maintaining fermentative metabolism is the major pathway under anaerobic conditions [12]. Alcohol dehydrogenase (ADH, EC 1.1.1.1), a fermentative enzyme, functions in the production of acetaldehyde and ethanol, thereby concomitantly regenerating NAD^+^ for glycolysis. A previous study reported that the *Adh* gene was expressed in anaerobic conditions and predominantly in the root [13]. Komatsu et al. [13] and Komatsu et al. [14] studied the transcriptome and proteomes in soybean. In a flooding experiment, it was found that genes associated with alcohol fermentation were significantly upregulated by more than 10,000-fold after 12 h compared to the control. In transgenic soybean, the introduced *GmADH2* gene induced glycolysis and alcohol fermentation, thereby improving germination under flooding stress [13,14]. In addition, the accumulation of toxic metabolites, including reactive oxygen species (ROS), ethanol, and acetaldehyde, may cause cellular damage and accelerate seed deterioration [1,10]. A previous study reported that soybean and turfgrass germination were inhibited in the presence of alcohol [1,15]. Therefore, recent studies have proved that changes in ADH activity play a vital role in flooding tolerance. Understanding the flood–tolerance mechanisms involved in the germination stage is important for improving soybean production and developing varieties suitable for flooded areas. Although the role of ADH in flooding tolerance has been documented in vegetative organs, its function during seed germination under anaerobic conditions remains poorly understood. Most previous studies compared only tolerant and susceptible genotypes without including a moderate group. Also, within the same tolerant group, individual genotypes may survive on distinct survival mechanisms; however, within-group diversity remains largely unexplored.

This study examined imbibition patterns, electrical conductivity, ADH activity, and ethanol accumulation across 15 soybean genotypes by seed soaking to simulate flooding stress, classified into three tolerant groups, at multiple time intervals. We hypothesized that the tolerant soybean group would demonstrate significantly higher ADH activity and lower electrical conductivity compared with the other groups. Moreover, each group consisted of five varieties, which enabled us to examine whether germination under flooding was attributable to a shared tolerance mechanism. The aim of this study was to elucidate the physiological responses in soybean by soaking seeds under laboratory conditions to simulate flooding stress. The knowledge gained from this study will deepen understanding of flooding tolerance among soybean groups and provide natural genetic resources for the soybean breeding program.

## 2. Results

### 2.1. Seed Imbibition Curve and Electrical Conductivity

**Figure 1:**
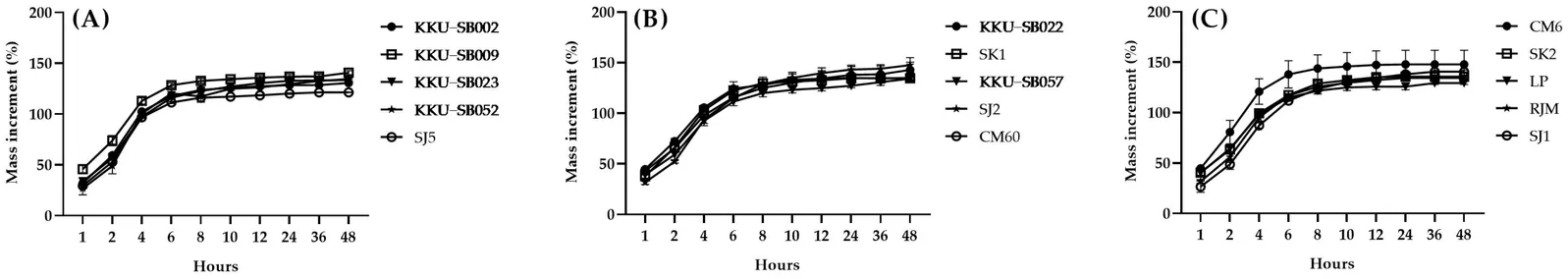
Water uptake curves of fifteen soybean varieties under exposure to different durations of water soaking: (**A**) tolerant group (**B**), moderate group, and (**C**) susceptible group. The values are means ± SE.

The results showed that rapid water uptake occurred between 1 and 6 h after seed imbibition. Overall, there was no difference in the imbibition curve between groups. The mass increment increased by approximately 100% relative to the initial weight after 4 h. This indicated that water rapidly enters the permeable seed coat, thereby increasing the seed coat’s volume. After 6 h, the water uptake curve entered the stationary phase or slightly increased water uptake, where physiological activities occurred (Figure 1A–C, Table S1).

**Figure 2:**
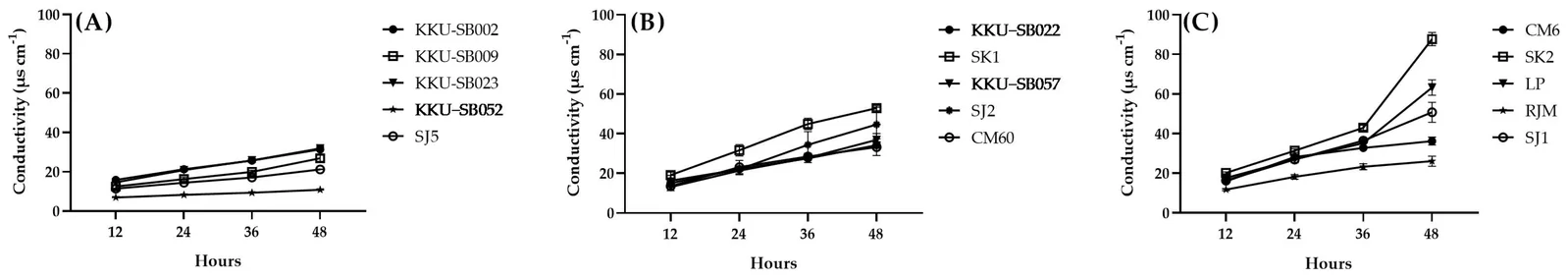
Effects of different durations of water soaking on conductivity from seed exudates in 15 soybean varieties: (**A**) tolerant group, (**B**) moderate group, and (**C**) susceptible group. The values are means ± SE.

Electrical conductivity (EC) was measured from seed exudate in each sample, and this trait can be used to discriminate between groups in terms of flooding tolerance based on germination. EC in the tolerant group tended to increase slightly in flooding intervals compared to the moderate and susceptible groups. The EC values in the tolerant group at 12 h ranged between 6.96 and 15.84 µs cm^−1^. KKU–SB052 showed a significant increase in EC after 48 h of water soaking (Figure 2A). This variety showed vigorous performance of the seeds when compared within and between groups (Figure 2A, Table 1). In the moderate group, the conductivity gradually increased with longer exposure to stress. An increase in EC was observed between 36 and 48 h after stress. SK1 showed significantly increased EC compared with the other varieties in its group and the tolerant group (Figure 2B). In susceptible groups, the increase in EC was higher than in moderate and tolerant groups. The significant increase in EC values in susceptible groups was observed 24 h after exposure to water soaking. SK2 had the highest EC values among all varieties (Figure 2C). Although RJM was classified in the susceptible group, the change in EC value in 12–48 h slightly increased (11.75–26.25 µs cm^−1^), and the values were close to KKU–SB009 (12.54–26.84 µs cm^−1^) (Figure 2C, Table 1).

### 2.2. Alcohol Dehydrogenase Activity (ADH)

**Figure 3:**
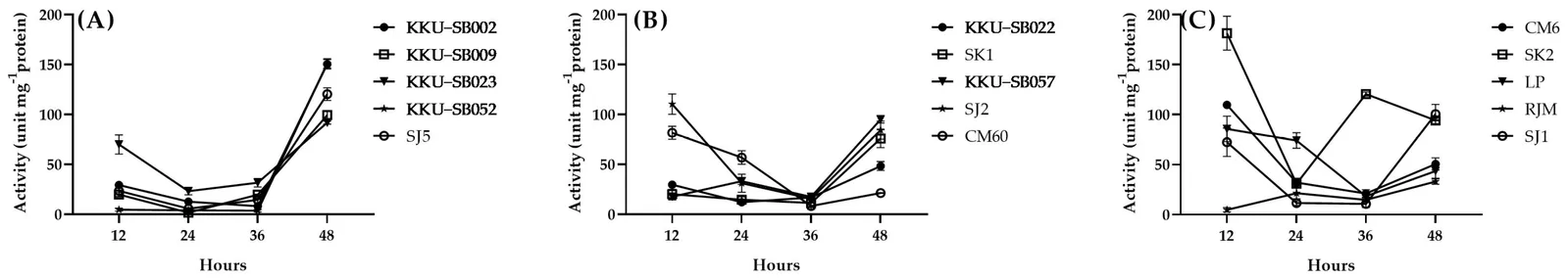
Effects of different durations of water soaking on ADH activities in cotyledon tissues in 15 soybean varieties (**A**) tolerant group, (**B**) moderate group, and (**C**) susceptible group. The values are means ± SE.

In cotyledon tissue, an increase in ADH activity was observed across all soybean groups after 12 h of water soaking (Figure 3). ADH activity tended to increase in the susceptible group (Table 2). This was 4–5 times higher than in other groups within the same exposure stress period. The highest ADH activity was observed in SK2, with 181.47 unit mg^−1^protein (Figure 3C). In contrast, the activities in the tolerant and moderate groups were approximately 20 unit mg^−1^ protein (Figure 3A,B, Table 2). This suggests that ADH activity responded in a different pattern after exposure to stress for different hours. The ADH activities in 24–36 h fluctuated and could not discriminate between groups. Interestingly, the activities in the tolerant and moderate groups continued to increase over 48 h of water soaking. ADH in the moderate group ranged from 20 to 95 units mg^−1^ protein. CM60 was the only variety that decreased ADH activity (21.44 units mg^−1^protein). In the tolerant group, ADH activities were induced in all varieties within 48 h, whereas in the other group, they were not (Figure 3A, Table 2).

**Figure 4:**
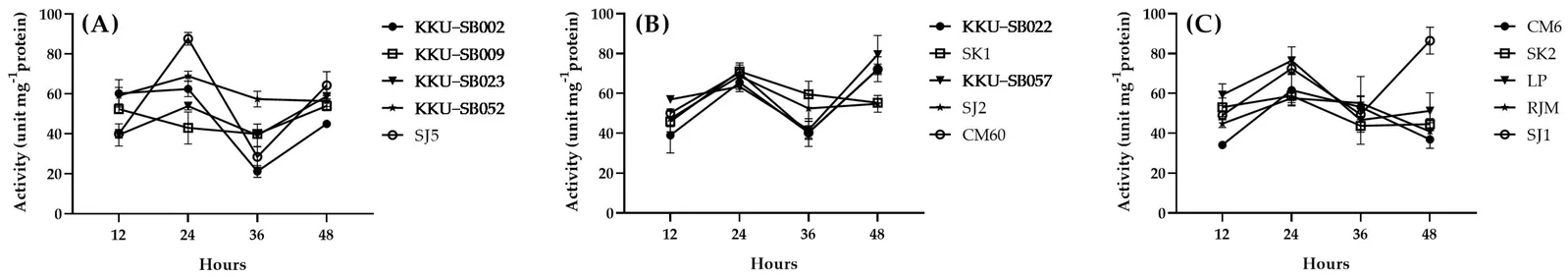
Effects of different durations of water soaking on ADH activities in embryonic tissues in 15 soybean varieties: (**A**) tolerant group, (**B**) moderate group, and (**C**) susceptible group. The values are means ± SE.

Overall, ADH enzyme activity in embryonic tissues fluctuated during exposure to stress. At 24 h, SJ5 showed the highest activity among the tolerant group, whereas in the susceptible group, ADH activity tended to decline at 48 h after stress, with the exception of SJ1, which increased in ADH activity (Figure 4, Table 3).

In comparison, ADH activity in both tissues showed different responses to water soaking for different durations (Figure 3 and Figure 4). ADH in cotyledon tissue plays a vital role that contributes to survival in soybean. Particularly, this enzyme was induced after 12 h of water soaking in susceptible groups and then dropped, while it was induced at 48 h in tolerant groups.

### 2.3. Ethanol Accumulation

**Figure 5:**
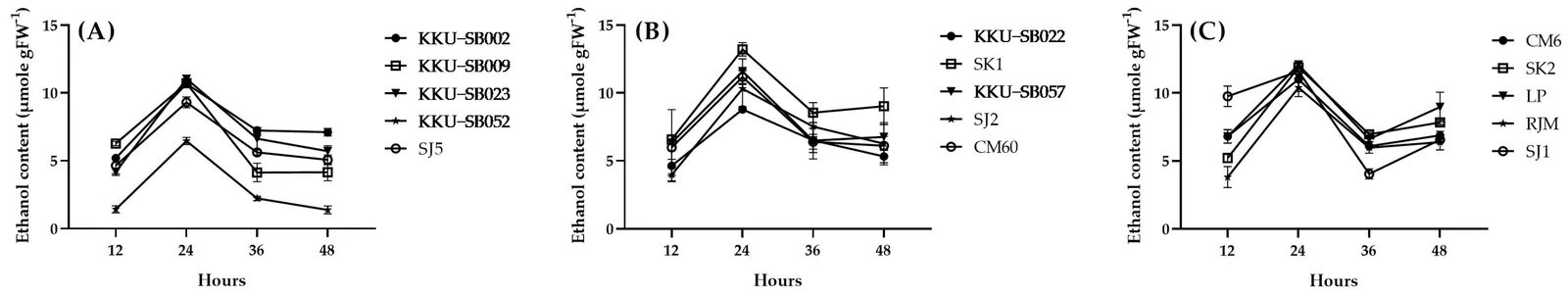
Effects of different durations of water soaking on ethanol content from seed exudates in 15 soybean varieties: (**A**) tolerant group, (**B**) moderate group, and (**C**) susceptible group. The values are means ± SE.

**Figure 6:**
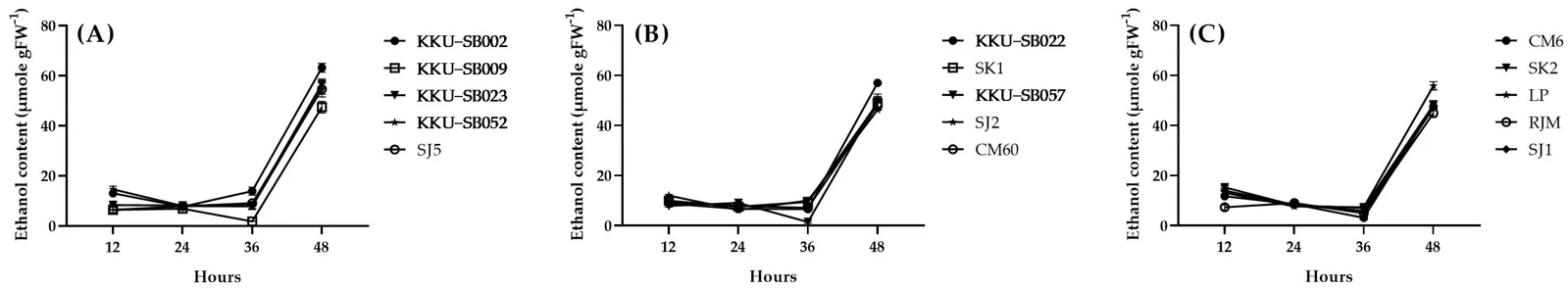
Effects of different durations of water soaking on ethanol content from seed tissues in 15 soybean varieties: (**A**) tolerant group, (**B**) moderate group, and (**C**) susceptible group. The values are means ± SE.

Ethanol content measured in both seed exudates (Figure 5, Table 4) and tissues (Figure 6, Table 5) indicated that ethanol levels could not effectively be parameterized to distinguish among soybean tolerance groups, as the values overlapped across groups. In addition, ethanol accumulation in tissues sharply increased at 48 h in all soybean groups compared to previous hours (Figure 6). Interestingly, KKU–SB052, which is categorized in the tolerance group, released less ethanol into the seed exudate compared with other genotypes in the same group (Figure 5A).

### 2.4. Hierarchical Clustering

**Figure 7:**
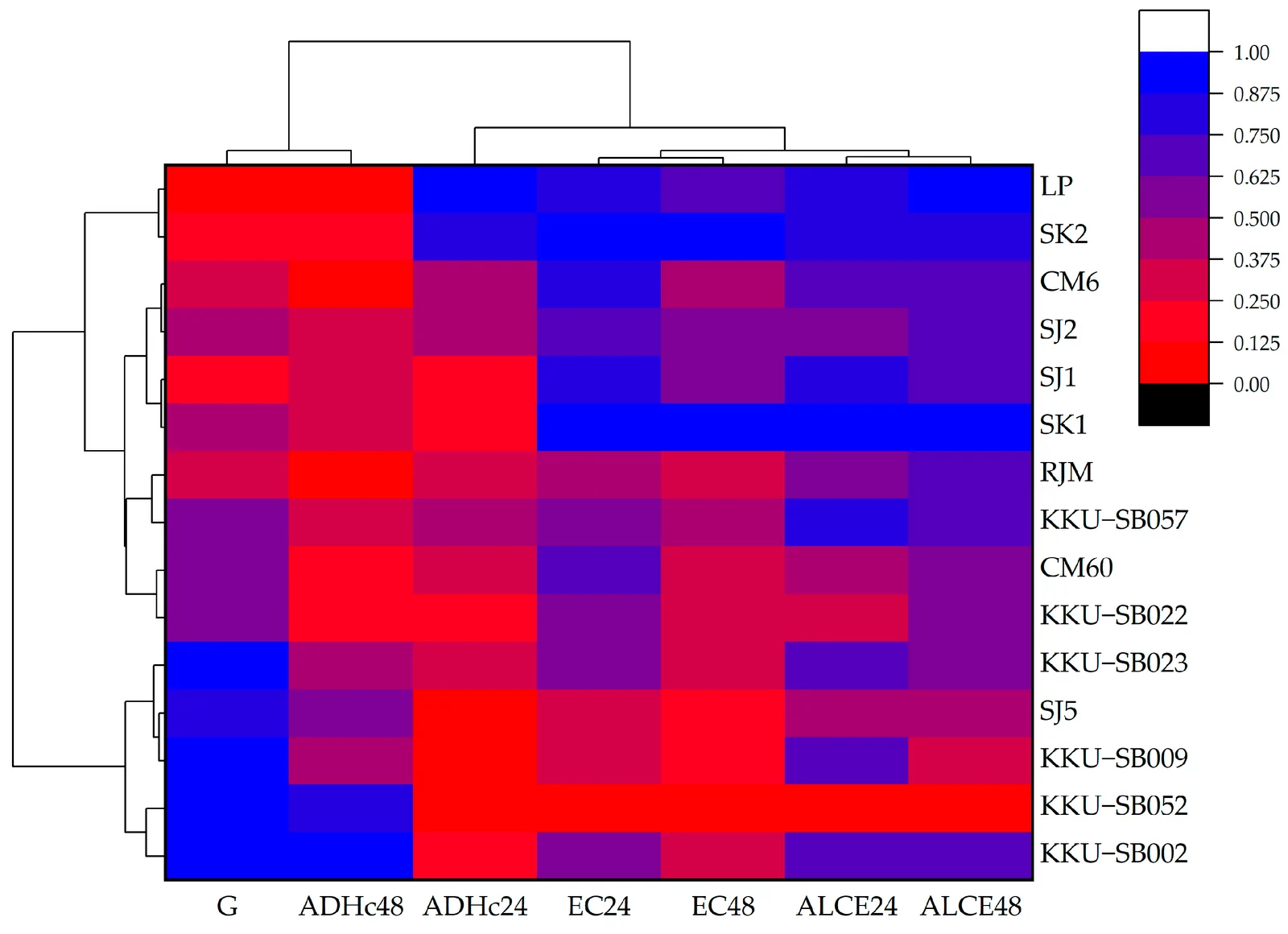
Hierarchical clustering heatmap and dendrogram of different soybean varieties exposed to water soaking for different durations. Each column represents a parameter measured in this study, and each row represents a variety. Blue indicates that the measured parameter was at a high level, and red indicates that the measured parameter was at a low level. (Abbreviations: G—germination percentage at 24 h after stress (preliminary screening results), ADHc24 and ADHc48—alcohol dehydrogenase activity in cotyledon tissues, EC24 and EC48—electrical conductivity in seed exudates, and ALCE24 and ALCE48—alcohol content in seed exudates; the numbers 24 and 48 represent hours of water soaking).

The response patterns of soybean to different periods of flooding stress and varieties were explained using a hierarchical heatmap. Hierarchical clustering was performed based on the germination percentage from the preliminary study. Overall, the cluster showed that the physiological responses differed within groups. The results from this study showed that EC (Figure 2), ADH activity in the cotyledon (Figure 3), and alcohol content in seed exudate (Figure 5) at 24 and 48 h differed. Therefore, these traits were used to further analyze with hierarchical clustering. Figure 7 used cluster analysis to provide an explanation of physiological patterns. The figure also showed that although high germination was found within the group, each variety used different responses to survive during water soaking.

The vertical clusters were grouped by measured traits, and the horizontal clusters by cultivars. The clustering results were divided into four distinct patterns. The first cluster was divided into two sub–clusters. KKU–SB002 and KKU–SB052, the tolerant group, showed similar patterns with high germination and ADH activity at 48 h and low EC at 24 h. KKU–SB052 had particularly low ethanol in seed exudate. This can be interpreted as indicating that this soybean variety responds to flooding stress after 48 h. Therefore, this indicated that the potential for germination in the tolerant group was associated with membrane maintenance and a delay in inducing ADH activity during water soaking. The second cluster, KKU–SB023, SJ5, and KKU–SB009 varieties, responded to stress faster than KKU–SB002 and KKU–SB052 at 24 h of water soaking, with a slight increase in EC and ethanol accumulation. The moderate and susceptible groups showed similar patterns. The third cluster included both moderate and susceptible varieties and was divided into two sub-clusters. The first sub-cluster is CM6, SJ2, SJ1, and SK1. These clusters shared the same response of low ADH activity with increasing EC and ethanol accumulation. On the other hand, the second sub-cluster, including RJM, KKU–SB57, CM60, and KKU–SB22, was similar to the first sub-cluster except in EC and ethanol accumulation.

Finally, cluster LP and SK2 tended to increase ADH activity at 24 h with high EC and ethanol. The clustering results suggested that EC and ADH activities can be separated from those of the tolerant group. Overall, these physiological traits can be used as potential indicators for classifying soybean genotypes based on their response and germination (Figure 3A).

## 3. Discussion

In this study, the imbibition patterns of all soybean groups showed a rapid increase in water uptake during the early phase, followed by a plateau as imbibition reached equilibrium (Figure 1). In the tolerant group, the imbibition curves among soybean genotypes showed low variation, indicating that these genotypes absorbed water efficiently under flooding conditions. In contrast, the moderate and susceptible groups exhibited greater variation in imbibition curves within each group, with SJ1 showing the lowest mass increment under water soaking, indicating that prolonged waterlogging severely disrupted water absorption and germination in susceptible genotypes. The results of this study indicated that solute leakage into the surrounding medium commonly occurs during seed imbibition; this leakage reflects the degree of membrane damage. The tolerant group showed only a slight increase in EC during the water soaking duration compared with the other groups, suggesting that tolerant genotypes can maintain membrane integrity under stress. This is consistent with findings reported by Zhou et al. [1] and Sharmin et al. [16], in which EC was negatively correlated with germination rate [1,16]. Koizumi et al. [17] studied the role of the seed coat in imbibing soybean seeds. The results found that when the testa was removed from the dry seeds before imbibition, the cotyledons were severely damaged within 1–5 h of water uptake [17]. However, some soybean varieties in the susceptible group were germinated; they produced a higher proportion of abnormal, stunted seedlings, and the seed coat separated from the cotyledon tissues (Figure S1). These structural abnormalities may contribute to reduced germination under water soaking. Our results suggest that differences in germination percentage under flooding stress are negatively correlated with EC during imbibition (Table S1). Previous studies in field pea also reported that electrical conductivity was strongly associated with waterlogging tolerance in the recombinant inbred line population. Testa integrity can be effectively used for indirect selection in waterlogging tolerance in pea and sudangrass [18,19]. However, the internal structure and water movement between the seed coat and the cotyledon area during imbibition should be further studied.

Flooding stress in soybeans causes oxygen deprivation and inhibits respiration. This condition not only inhibits the conversion of triglycerides to sugar, affecting the mobilization of seed reserves, but also represses gibberellic acid biosynthesis during seed imbibition [1]. This study used seed soaking to mimic flooding. The results showed that ADH activity in cotyledon tissue was upregulated at 48 h of water soaking in the tolerant group; however, this enzyme responded earlier, at 12 h post-exposure, in the susceptible group (Figure 3). ADH activity in the moderate group was also induced at 12 h after stress; however, the value was lower than that of the susceptible group. In contrast, the early response in the susceptible soybean group implied that susceptible genotypes were exposed to water soaking (Figure 3C). A similar trend was found in the study by Rizal and Karki [20]. Tamahomare (susceptible cultivar to flooding stress) showed higher ADH activity during exposure to the stress for 6 h, while Peking (tolerant cultivar to flooding stress) showed lower ADH activity within the same period of exposure [20]. An increase in ADH activity contributes to survival and the overcoming of energy scarcity by fermenting carbohydrates to maintain ATP production in the absence of oxygen [21,22]. Early induction of ADH activity in the susceptible soybean group suggested that it altered its metabolism, which may be involved in anaerobic metabolism (Figure 4C). In this study, ADH activity in the embryonic axis did not differ in pattern across groups. This suggests that ADH in cotyledon tissues plays an important role rather than in embryonic tissue in contributing to the survival of the seed under seed soaking. During seed germination, respiration occurs in early seed imbibition to produce the energy for germination. Nutrients in the seed are mobilized from the storage reserves [23]. According to the results in this study, improved ADH in storage tissue in the cotyledons contributes to higher germination performance.

During flooding stress, ADH regenerates NAD^+^ by reducing acetaldehyde to ethanol. Toxic substances produced during oxygen deprivation cause seed deterioration. The previous studies in soybean cv. Essex found that acetaldehyde and ethanol production increased under anaerobic conditions; additionally, a low rate of oxygen uptake was observed in the embryo axis of deteriorated seed [24]. In this study, the results showed that ethanol cannot distinguish between soybean groups (Figure 5 and Figure 6). Ethanol accumulated in plant cells is considered a toxic substance and could interfere with germination. This finding was consistent with the ethanol test across different vigor soybeans. A previous study reported the association between EC and seed respiration. It was observed that high-vigor seeds release ethanol more slowly than low-vigor seeds [22]. In this study, the EC values for each soybean group showed differences in the increase over a single hour when exposed to different periods of stress, which vary according to their ability to tolerate water soaking. The results suggested that membrane integrity in susceptible group soybeans was easily damaged, although the ethanol content was found in the same amount as in the moderate and tolerant soybean groups. RJM was the only variety that showed the lowest EC among the susceptible group (Figure 2C). Ethanol accumulation and ADH activity in cotyledon tissue tended to increase in this variety. This implied that other physiological responses might contribute to survival under water soaking. Further study of aerobic metabolism should be conducted. Also, KKU–SB052response to flooding that differs from other varieties may be from its seed size and seed coat color. Small seeds were considered to be more adaptable in saturated soil compared to large seeds because they have a larger surface area to volume ratio, which allows water to diffuse faster from the seed coat to the embryo [25].

**Figure 8:**
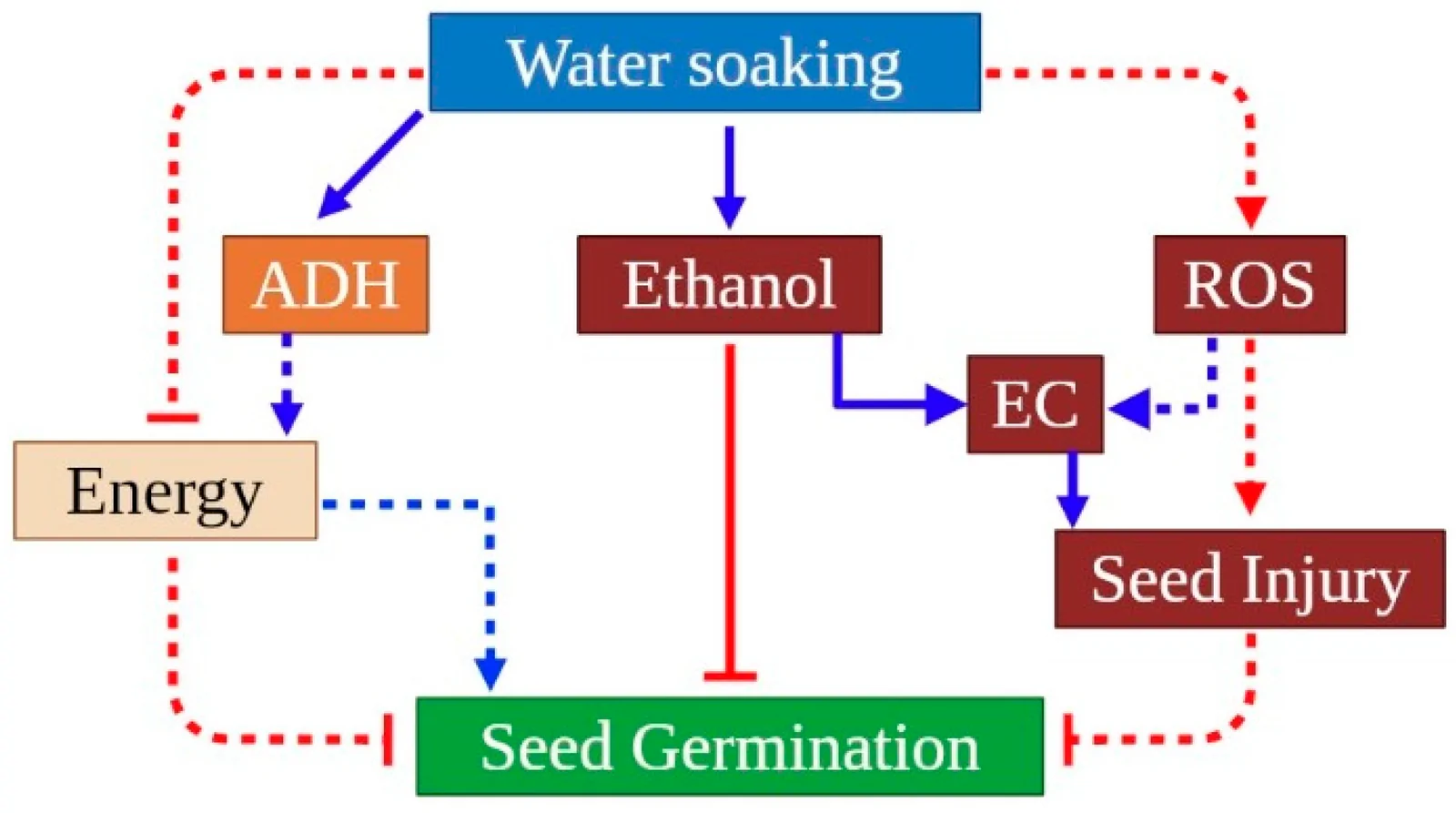
Proposed mechanism of flooding stress response during germination in this study. (Abbreviations: ADH—alcohol dehydrogenase; EC—electrical conductivity; ROS—reactive oxygen species.) The blue arrow represents an increase, and the red arrow represents a decrease. The solid line represents the results obtained from this study, and the dashed line represents the hypothetical mechanism-related measured parameter to flooding stress in this study.

The possible mechanism of flooding tolerance in the tolerant soybean group, as suggested by this study, is illustrated in Figure 8. During seed exposure to water soaking, several physiological responses are triggered. The presence of excess ethanol damages seeds and reduces vigor. As a result, cell membrane integrity is damaged, leading to higher electrical conductivity in seed exudates. However, an alternative pathway in anaerobic metabolism is activated. ADH in cotyledon tissues is induced to provide energy for germination by regenerating NAD^+^ through the glycolysis pathway. In this study, KKU–SB052 showed a high germination percentage under flooding stress, low ethanol production and electrical conductivity, and high ADH activity after prolonged flooding stress. However, the moderate soybean group might use these proposed mechanisms at a lower level of response during the 36 h of water soaking.

## 4. Materials and Methods

### 4.1. Plant Materials

Seventy-four soybean germplasms were obtained from the Plant Breeding Research Center for Sustainable Agriculture, Khon Kaen University (KKU), Thailand. The soybean materials used in this study were originally obtained from the Thai DOA and the NIAS World Soybean Core Collection in 2014 and have subsequently been maintained at KKU, Thailand. Local identifiers (KKU–SBxxx) were assigned to the materials maintained at KKU. The corresponding original cultivar/line names and accession information are provided in Table S2. All soybean germplasms were cultivated from December 2022 to March 2023 at the Agronomy Field Crop Station. The obtained seeds were dehumidified by sun drying.

### 4.2. Preliminary Screening

All seeds were evaluated for flooding tolerance under laboratory conditions as described below. The preliminary screening of each soybean variety was conducted with three replicates, each consisting of 25 seeds. Twenty-five soybean seeds were imbibed in 100 mL beakers with 3 cm of distilled water above the soybean surface. The seeds were exposed to flooding stress for 24 h, and the seeds were moved to germinate in a germination chamber using the between-paper method with an alternative light–dark condition (12 h), temperature (25 and 30 °C), and 70% humidity. The data was recorded 8 days after planting. The germination percentage thresholds for classifying tolerance levels were defined into three ranges specifically for this study as follows: tolerant (≥70%), moderately tolerant (40–69%), and susceptible (≤39%). Five soybean varieties were selected to represent each group. The criteria for selecting soybean varieties in each group were based on the range in similar seed size and yellow seed coat color of SJ5 and CM60 (Thailand commercial cultivar) to minimize interference from other factors (except in KKU-SB052), which showed high value in germination but small seed size and green seed coat color; see Figure S2 for more information. The selected varieties for each group were listed in Table 6.

### 4.3. Experimental Design

Fifteen soybean varieties based on flooding tolerance were used in this study (Table 6). The experimental design was carried out in laboratory conditions using a completely randomized design (CRD) with three replicates. Flooding conditions were similar to those mentioned above in the previous preliminary study. After the seeds were soaked for 12, 24, 36, and 48 h, the samples were collected for further investigation.

### 4.4. Seed Imbibition Curve and Conductivity Quantification

Seed weight was measured by a modified method described by Viana et al. [26]. The seeds were weighed before imbibition and at 1, 2, 4, 6, 8, 10, 12, 24, and 36 h, and the experiment was terminated at 48 h. In this study, the seed imbibition curve was expressed as relative weight (%). The parameter was calculated as mass increment (%) = [(M_t_ − M_i_)/M_i_] × 100, where M_i_ = initial fresh mass of the sample and Mt = mass of the sample measured at time (hours) [26].

For conductivity measurements, fluid from each seed imbibed at different hours (12, 24, 36, and 48 h) was collected [1]. The seed exudates were directly measured using a Mettler–Toledo conductivity meter and expressed in units of µs/cm.

### 4.5. Alcohol Dehydrogenase Activity

In this study, cotyledon and embryonic tissues were investigated to understand their responses to anaerobic conditions across different flooding periods. Alcohol dehydrogenase (ADH) activity was assayed using the method described by Fukao et al. [27], with modifications to the reaction volume. Cotyledon and embryo axis tissue (50 mg) imbibed for different hours were separately excised and homogenized in a pre-chilled mortar and pestle by adding 0.4 mL extraction buffer (containing 100 mM Tris-HCl pH 9.0, 20 mM MgCl_2_, and 0.1% (*v*/*v*) 2-Mercaptoethanol). The crude extract was then centrifuged, and the supernatant was collected for the ADH assay. The reaction mixture (final volume was 2 mL) contained 100 µL of extract, 50 mM Tris-HCl (pH 9.0), and 1 mM NAD^+^. A total of 100 µL of ethanol was added to initiate the reaction. NAD^+^ reduction was monitored at 340 nm using a spectrophotometer for 2 min E = 6.22 mM^−1^ cm^−1^), where E is the molar extinction coefficient [27]. Protein was assayed using the method described by Bradford (1976) and monitored at 595 nm, with Bovine Serum Albumin as the standard [28].

### 4.6. Ethanol Determination

Ethanol is a toxic substance that damages cells and compromises cell integrity. Ethanol in seed tissue and seed exudates was determined by the method described by Fukao et al. [27]. Imbibed seed tissue and seed exudates collected at different time points were analyzed. About 50 mg of seed tissue was ground with pre-cooled 0.2 mL of 5 M HClO_4_ and centrifuged at 4 °C for 20 min at 16,000× *g*. The supernatant was collected, and 100 µL of the extract was neutralized with 0.25 mL of 1 M K_2_CO_3_. After neutralization, the white precipitate was removed by centrifugation. The supernatant obtained from seed tissue and exudate (100 µL) was used for the ethanol assay (900 µL) containing 100 mM Tris-HCl pH 9.0, 0.6 mM NAD^+^, and 40 units of ADH [27]. The samples were incubated at 25 °C for 1 h. The reaction was monitored at 340 nm using a spectrophotometer. Ethanol was used as the standard curve for ethanol content calculation.

### 4.7. Statistical Analysis

The data were statistically analyzed for each parameter using one-way analysis of variance (ANOVA) to test for significant differences among varieties. The mean comparison was performed using Least Significant Difference (LSD) at *p* ≤ 0.05 [29]. Pearson correlation analysis was performed to assess the relationships among traits in each flooding period. The statistical analysis was performed using the Statistix 10 program. Hierarchical clustering analysis was conducted using Origin 2022 to group soybean varieties based on various traits. The data were normalized using the rescaling method (0–1). Clustering was performed using Pearson correlation distance type and the furthest neighbor cluster.

## 5. Conclusions

Soybean seeds alter metabolism under flooding stress by increased ADH in cotyledon tissue at 12–24 h in the moderate and susceptible groups. In contrast, ADH was induced in the tolerant group after 48 h. Prolonged exposure to stress increased seed injury, as indicated by increased conductivity in seed exudates and ethanol accumulation in tissue. ADH activity plays an important role in the continued production of energy during germination. The tolerant soybean group maintained a high germination percentage, as indicated by low ethanol accumulation in exudates, preserved membrane integrity, and maintained ADH activity at 48 h; however, the susceptible group showed an early response in ADH activity and higher conductivity. Therefore, ADH activity in cotyledon tissue and electrical conductivity should be used as criteria to evaluate flooding tolerance.

KKU–SB052 variety is recommended for use as a natural genetic source for a potential crossing line for the transfer of these traits in soybean breeding programs. In addition, the results of this study can serve as a potential indicator for assessing water soaking or flooding tolerance. These potential indicators can be used for further study as criteria for selecting, adding, or gene pyramiding in soybean breeding programs.

## Figures and Tables

**Table 1:** Effects of different durations of water soaking on electrical conductivity from seed exudates in 15 soybean varieties.

Groups	Varieties	Electrical Conductivity (µs cm^−1^)							
		12 h		24 h		36 h		48 h	
Tolerant	KKU–SB002	15.84 ± 0.30	BC	21.32 ± 0.58	DE	25.70 ± 0.82	CD	31.37 ± 0.68	EFG
	KKU–SB009	12.54 ± 0.96	DE	16.26 ± 1.00	FG	19.99 ± 0.80	EF	26.84 ± 1.52	FGH
	KKU–SB023	14.59 ± 0.37	BCD	20.90 ± 0.62	DE	25.75 ± 0.84	CD	31.90 ± 0.87	EFG
	KKU–SB052	6.96 ± 0.06	F	8.32 ± 0.04	H	9.41 ± 0.08	G	10.86 ± 0.14	I
	SJ5	11.48 ± 0.26	E	14.46 ± 0.40	G	17.08 ± 0.31	F	21.24 ± 0.15	H
Moderate	KKU–SB022	15.03 ± 0.19	BCD	22.11 ± 0.21	D	27.56 ± 0.52	C	34.22 ± 0.78	EF
	SK1	19.15 ± 0.86	A	31.65 ± 1.63	A	44.86 ± 1.62	A	52.88 ± 0.74	C
	KKU–SB057	13.10 ± 1.06	DE	21.37 ± 1.19	DE	27.80 ± 1.33	C	36.84 ± 1.86	E
	SJ2	16.28 ± 1.55	B	22.00 ± 0.51	D	34.40 ± 3.81	B	44.61 ± 5.04	D
	CM60	13.55 ± 0.32	CDE	23.10 ± 1.93	D	28.46 ± 0.87	C	33.17 ± 2.43	EFG
Susceptible	CM6	15.93 ± 0.94	BC	28.23 ± 0.78	BC	32.78 ± 0.41	B	36.24 ± 1.95	E
	SK2	20.23 ± 0.48	A	31.36 ± 0.94	AB	42.94 ± 1.25	A	87.72 ± 3.35	A
	LP	17.62 ± 0.77	A	27.07 ± 1.77	C	35.18 ± 1.45	B	63.28 ± 3.87	B
	RJM	11.75 ± 0.43	E	18.22 ± 1.15	EF	23.31 ± 1.53	DE	26.25 ± 2.43	GH
	SJ1	16.40 ± 0.20	B	26.99 ± 1.64	C	36.49 ± 1.15	B	50.76 ± 5.07	CD
	Mean	14.82		22.23		28.78		39.21	
	CV (%)	10.90		8.68		8.45		11.46	
	F-test	**		**		**		**	
	LSD	2.69		3.22		4.06		7.50	

Data (means ± SE) followed by different letters within the same column indicate a significant difference according to the LSD test (** significant at *p* ≤ 0.01).

**Table 2:** Effects of different durations of water soaking on alcohol dehydrogenase activity from cotyledon tissues in 15 soybean varieties.

Groups	Varieties	Alcohol Dehydrogenase Activity (Unit mg^−1^Protein)							
		12 h		24 h		36 h		48 h	
Tolerant	KKU–SB002	29.43 ± 1.76	D	12.68 ± 0.33	DEF	8.22 ± 0.27	EF	150.35 ± 4.81	A
	KKU–SB009	19.96 ± 2.05	DE	1.71 ± 0.06	F	19.73 ± 0.45	C	99.44 ± 4.30	C
	KKU–SB023	70.03 ± 9.56	C	23.21 ± 3.70	CD	31.63 ± 4.23	B	91.91 ± 1.55	CD
	KKU–SB052	4.73 ± 1.46	E	4.02 ± 1.13	EF	3.72 ± 1.27	F	151.23 ± 4.62	A
	SJ5	23.61 ± 6.10	DE	5.78 ± 1.01	EF	14.56 ± 2.91	CDE	120.47 ± 6.34	B
Moderate	KKU–SB022	29.83 ± 1.89	D	12.22 ± 1.82	DEF	16.92 ± 2.13	CD	48.75 ± 4.43	EF
	SK1	20.26 ± 4.91	DE	14.69 ± 2.84	DE	11.38 ± 4.31	DEF	75.88 ± 8.94	D
	KKU–SB057	17.71 ± 3.18	DE	33.22 ± 3.06	C	17.65 ± 3.09	CD	95.13 ± 3.72	C
	SJ2	110.44 ± 10.17	B	31.26 ± 8.99	C	15.67 ± 1.32	CDE	84.36 ± 9.00	CD
	CM60	81.75 ± 6.54	C	56.99 ± 6.77	B	8.45 ± 3.35	EF	21.44 ± 0.33	G
Susceptible	CM6	109.73 ± 1.51	B	32.07 ± 4.38	C	21.06 ± 3.81	C	50.60 ± 6.07	E
	SK2	181.47 ± 16.91	A	30.85 ± 2.01	C	120.59 ± 1.96	A	94.32 ± 0.64	C
	LP	85.75 ± 12.62	C	74.15 ± 7.79	A	18.38 ± 0.94	CD	43.58 ± 7.23	EF
	RJM	4.80 ± 2.24	E	21.45 ± 5.66	CD	14.74 ± 2.14	CDE	33.06 ± 2.63	FG
	SJ1	72.43 ± 14.23	C	11.50 ± 2.32	DEF	10.69 ± 3.43	DEF	100.61 ± 9.70	C
	Mean	57.46		24.39		22.23		84.05	
	CV (%)	21.97		30.96		21.06		11.78	
	F-test	**		**		**		**	
	LSD	23.31		12.59		7.81		16.52	

Data (means ± SE) followed by different letters within the same column indicate a significant difference according to the LSD test (** significant at *p* ≤ 0.01).

**Table 3:** Effects of different durations of water soaking on alcohol dehydrogenase activity from embryonic tissues in 15 soybean varieties.

Groups	Varieties	Alcohol Dehydrogenase Activity (Unit mg^−1^Protein)							
		12 h		24 h		36 h		48 h	
Tolerant	KKU–SB002	36.03 ± 6.88	C	62.48 ± 3.88	BCD	21.20 ± 2.96	D	44.92 ± 0.40	E
	KKU–SB009	52.39 ± 2.22	AB	42.92 ± 8.01	E	39.84 ± 1.74	BCD	53.99 ± 2.43	CDE
	KKU–SB023	39.42 ± 5.52	BC	53.83 ± 1.87	DE	39.17 ± 5.70	BCD	58.76 ± 1.31	CDE
	KKU–SB052	59.10 ± 4.13	A	68.90 ± 2.50	BCD	57.35 ± 3.93	AB	56.36 ± 2.76	CDE
	SJ5	39.98 ± 2.12	BC	103.50 ± 3.15	A	28.45 ± 5.19	CD	64.27 ± 6.84	BCD
Moderate	KKU–SB022	38.99 ± 8.87	BC	65.48 ± 3.45	BCD	40.32 ± 6.96	BC	72.10 ± 2.61	ABC
	SK1	45.85 ± 5.12	ABC	71.03 ± 3.24	BCD	59.51 ± 6.64	A	55.30 ± 2.05	CDE
	KKU–SB057	56.95 ± 1.93	A	63.49 ± 2.57	BCD	41.71 ± 4.14	ABC	79.58 ± 9.44	AB
	SJ2	47.32 ± 4.03	ABC	68.70 ± 4.19	BCD	52.48 ± 6.43	AB	54.75 ± 4.22	CDE
	CM60	50.11 ± 2.08	ABC	70.29 ± 5.01	BCD	40.32 ± 3.42	BC	72.13 ± 6.21	ABC
Susceptible	CM6	34.15 ± 1.70	C	61.58 ± 7.91	BCD	53.09 ± 5.24	AB	51.36 ± 4.42	DE
	SK2	53.04 ± 4.67	AB	58.77 ± 3.42	CDE	43.70 ± 3.13	ABC	44.50 ± 2.78	E
	LP	59.33 ± 5.46	A	76.49 ± 6.89	B	46.65 ± 12.18	ABC	51.24 ± 9.01	DE
	RJM	44.78 ± 1.91	ABC	57.66 ± 3.46	CDE	55.08 ± 13.56	AB	72.81 ± 4.39	ABC
	SJ1	49.08 ± 1.83	ABC	72.48 ± 2.42	BC	49.48 ± 3.95	AB	86.49 ± 6.73	A
	Mean	47.10		66.51		44.56		61.24	
	CV (%)	20.61		15.93		25.17		18.93	
	F-test	*		*		*		*	
	LSD	16.19		17.67		18.70		19.33	

Data (means ± SE) followed by different letters within the same column indicate significant differences according to the LSD test (* significant at *p* ≤ 0.05).

**Table 4:** Effects of different durations of water soaking stress on alcohol content from seed exudates in 15 soybean varieties.

Groups	Varieties	Ethanol Content (µmole g FW^−1^)							
		12 h		24 h		36 h		48 h	
Tolerant	KKU–SB002	5.20 ± 0.23	BCD	10.68 ± 0.28	B–E	7.23 ± 0.23	BC	7.10 ± 0.28	B
	KKU–SB009	6.27 ± 0.15	BC	10.71 ± 0.17	B–E	4.14 ± 0.68	E	4.16 ± 0.62	F
	KKU–SB023	4.19 ± 0.25	D	11.05 ± 0.27	BCD	6.63 ± 0.80	BCD	5.71 ± 0.40	C–F
	KKU–SB052	1.43 ± 0.26	E	6.45 ± 0.26	G	2.23 ± 0.16	F	1.38 ± 0.29	G
	SJ5	4.64 ± 0.59	CD	9.31 ± 0.40	EF	5.61 ± 0.18	D	5.06 ± 0.36	EF
Moderate	KKU–SB022	4.63 ± 0.64	CD	8.78 ± 0.12	F	6.50 ± 0.52	BCD	5.31 ± 0.26	DEF
	SK1	6.57 ± 0.19	B	13.21 ± 0.29	A	8.55 ± 0.43	A	9.02 ± 0.78	A
	KKU–SB057	6.33 ± 1.41	B	11.60 ± 0.52	ABC	6.48 ± 0.78	BCD	6.76 ± 0.53	BCD
	SJ2	3.97 ± 0.29	D	10.31 ± 0.76	C–F	7.52 ± 0.55	AB	6.26 ± 0.90	CDE
	CM60	5.98 ± 0.50	BC	9.54 ± 1.61	DEF	6.40 ± 0.32	BCD	6.10 ± 0.40	CDE
Susceptible	CM6	6.80 ± 0.50	B	11.00 ± 0.90	B–E	6.08 ± 0.06	CD	6.88 ± 0.30	BC
	SK2	5.21 ± 0.34	BCD	11.90 ± 0.08	ABC	6.96 ± 0.16	BC	7.86 ± 0.16	AB
	LP	6.79 ± 0.49	B	12.08 ± 0.29	AB	6.61 ± 0.18	BCD	8.97 ± 1.08	A
	RJM	3.81 ± 0.77	D	10.39 ± 0.66	B–F	5.97 ± 0.41	CD	6.38 ± 0.57	B–E
	SJ1	9.75 ± 0.75	A	11.60 ± 0.20	ABC	4.05 ± 0.37	E	6.56 ± 0.11	B–E
	Mean	5.13		10.50		6.21		6.21	
	CV (%)	18.53		9.76		12.87		15.03	
	F-test	**		**		**		**	
	LSD	1.68		1.72		1.30		1.56	

Data (means ± SE) followed by different letters within the same column indicate a significant difference according to the LSD test (** significant at *p* ≤ 0.01).

**Table 5:** Effects of different durations of water soaking on alcohol content from tissues in 15 soybean varieties.

Groups	Varieties	Ethanol Content (µmole g FW^−1^)							
		12 h		24 h		36 h		48 h	
Tolerant	KKU–SB002	13.03 ± 0.34	AB	7.71 ± 0.25	BCD	13.89 ± 1.61	A	63.22 ± 1.76	A
	KKU–SB009	6.43 ± 1.16	G	6.92 ± 0.12	DEF	1.87 ± 0.04	H	47.44 ± 2.34	D
	KKU–SB023	8.34 ± 0.36	EFG	8.13 ± 0.38	ABC	8.15 ± 1.16	B–E	56.71 ± 1.97	B
	KKU–SB052	14.62 ± 1.20	A	7.96 ± 0.28	BC	7.80 ± 1.21	B–F	54.89 ± 2.24	BC
	SJ5	6.46 ± 0.16	G	7.93 ± 0.43	BCD	9.13 ± 0.65	BCD	54.62 ± 3.09	BC
Moderate	KKU–SB022	8.83 ± 0.96	EFG	6.68 ± 0.58	EF	6.61 ± 0.62	DEF	57.00 ± 0.31	B
	SK1	10.00 ± 1.36	CDE	7.56 ± 0.26	CDE	9.49 ± 1.10	BC	48.95 ± 2.65	D
	KKU–SB057	7.90 ± 0.49	EFG	9.13 ± 0.31	A	1.48 ± 0.42	H	49.63 ± 2.93	CD
	SJ2	12.05 ± 0.35	BC	6.22 ± 0.14	F	10.02 ± 1.12	B	46.22 ± 0.20	D
	CM60	9.46 ± 0.44	DEF	7.96 ± 0.39	BC	7.09 ± 1.21	C–F	48.18 ± 0.93	D
Susceptible	CM6	11.80 ± 0.96	BCD	8.59 ± 0.30	AB	3.15 ± 0.81	GH	47.72 ± 1.43	D
	SK2	15.30 ± 0.66	A	8.12 ± 0.18	ABC	7.04 ± 0.83	C–F	48.26 ± 1.71	D
	LP	13.17 ± 0.99	AB	7.70 ± 0.41	BCD	7.34 ± 1.55	B–F	55.86 ± 1.65	B
	RJM	7.34 ± 0.96	FG	9.04 ± 0.57	A	4.96 ± 0.67	FG	44.91 ± 1.55	D
	SJ1	13.96 ± 1.24	AB	8.39 ± 0.29	ABC	5.91 ± 0.37	EFG	47.06 ± 0.39	D
	Mean	10.34		7.83		7.00		51.69	
	CV (%)	14.14		7.78		24.73		6.39	
	F-test	**		**		**		**	
	LSD	2.50		1.02		2.86		5.47	

Data (means ± SE) followed by different letters within the same column indicate a significant difference according to the LSD test (** significant at *p* ≤ 0.01).

**Table 6:** Soybean varieties, seed coat color, seed size, and preliminary screening germination percentage after flooding for 24 h used in this study.

No.	Groups	Varieties	Seed Coat Color	Seed Size (mm)			Germination After WAter Soaking for 24 h (%)
				Length	Width	Thickness	
1	Tolerant	KKU–SB002	Yellow	8.85	7.60	5.80	96.67
2		KKU–SB009	Yellow	7.25	6.50	5.65	93.33
3		KKU–SB023	Yellow	7.45	6.30	5.20	95.00
4		KKU–SB052	Green	6.00	5.25	3.95	100.00
5		Sorjor5 (SJ5)	Yellow	8.05	6.40	5.05	81.67
6	Moderate	KKU–SB022	Yellow	7.05	5.70	4.55	56.67
7		Sukhothai1 (SK1)	Yellow	6.30	5.55	4.60	48.33
8		KKU–SB057	Yellow	8.35	6.90	5.85	63.33
9		Sorjor2 (SJ2)	Yellow	7.20	5.55	4.80	46.67
10		Chiang Mai 60 (CM60)	Yellow	6.90	6.10	5.30	51.67
11	Susceptible	Chiang Mai 6 (CM6)	Yellow	7.05	6.35	5.15	33.33
12		Sukhothai2 (SK2)	Yellow	6.95	6.30	5.25	16.67
13		Lopburi84–1 (LP)	Yellow	6.60	6.05	5.10	3.33
14		Rajamagala1 (RJM)	Yellow	7.80	6.15	4.95	28.52
15		Sorjor1 (SJ1)	Yellow	7.60	6.35	5.05	24.00

## Data Availability

Data are archived in W.J.’s lab and are available upon request.

## Supplementary Materials

The following supporting information can be downloaded at: https://www.mdpi.com/article/10.3390/plants15213302/s1, Figure S1: Preliminary study in soybean seedling evaluation after exposure to water soaking for 24 hand germination in a growth chamber for 7 days (**A**) KKU–SB052 (tolerance group) (**B**) CM60 (moderate group) and (**C**) SJ1 (susceptible group); Figure S2: Seeds of fifteen soybean varieties used in this study; Table S1: Effects of different periods of flooding stress on imbibition curve in 15 soybean varieties; Table S2: Original soybean names of soybean germplasms used in this study. A total of 74 accessions were evaluated in the preliminary screening, from which 15 accessions were selected for the experiments reported in the manuscript.

## Abbreviations

The following abbreviations are used in this manuscript:

ADH	Alcohol dehydrogenase
CM6	Chiang Mai 6
CM60	Chiang Mai 60
EC	Electrical conductivity
LP	Lopburi 84–1
RJM	Rajamagala1
ROS	Reactive oxygen species
SJ1	Sorjor1
SJ2	Sorjor2
SJ5	Sorjor5
SK1	Sukhothai1
SK2	Sukhothai2
