# Supplementary material for: Alcohol Dehydrogenase Activity and Electrical Conductivity of Seed Exudates as Potential Indicators of Flooding Tolerance in Soybean Groups During the Germination Stage
Source: Plants (Basel). 2026 Oct 26;15(21):3302. doi: 10.3390/plants15213302 (PMC13765933; doi:10.3390/plants15213302)
Supplement: Supplementary file 1 [file plants-15-03302-s001.zip › Supplementary data.pdf]

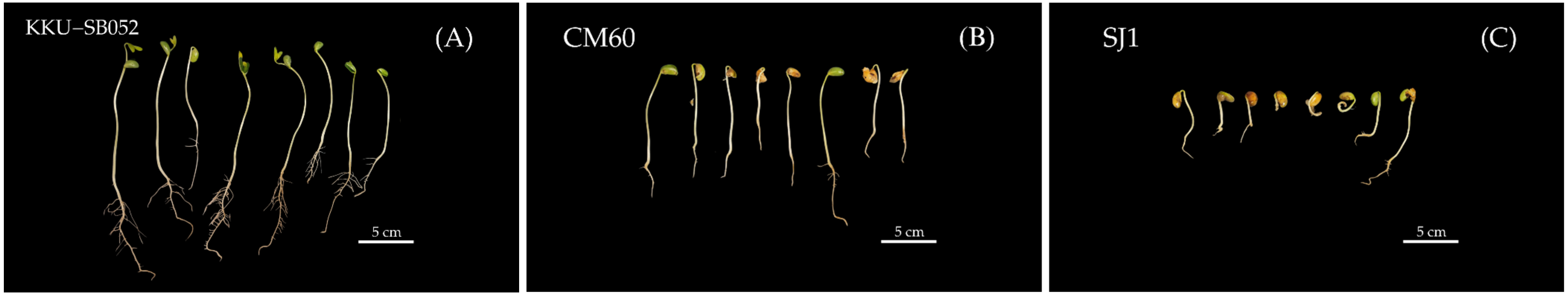

Figure S1. Preliminary study in soybean seedling evaluation after exposure to water soaking for 24 hand germination in a growth chamber for 7 days (**A**) KKU–SB052 (tolerance group) (**B**) CM60 (moderate group) and (**C**) SJ1 (susceptible group)

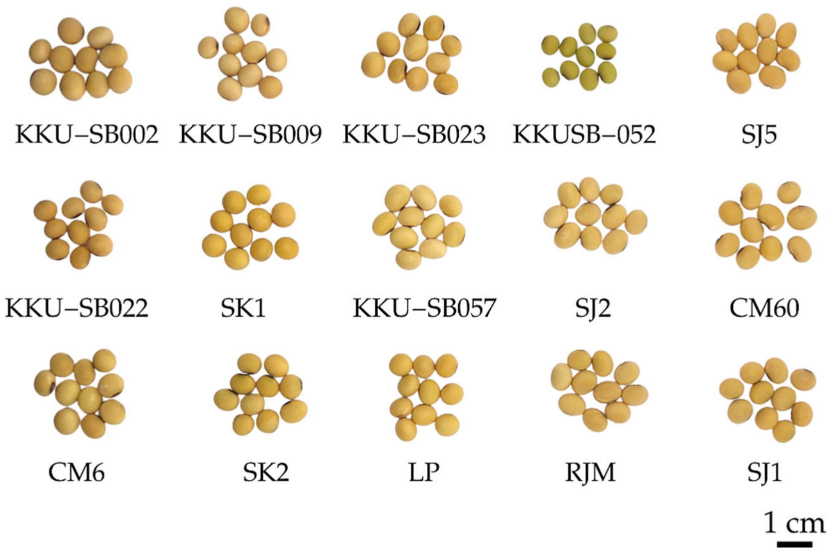

Figure S2. Seeds of fifteen soybean varieties used in this study

Table S1. Effects of different periods of flooding stress on imbibition curve in 15 soybean varieties

Groups	Varieties	Mass increment (%)													
		1 hours		2 hours		4 hours		6 hours		8 hours		10 hours		12 hours	
Tolerant	KKU−SB002	31.07	D-G	59.19	CDE	100.58	CD	116.28	CD	122.51	B-E	124.99	CDE	126.64	CDE
	KKU−SB009	49.36	A	77.41	A	115.02	AB	123.3	AB	133.99	AB	135.19	ABC	136.3	ABC
	KKU−SB023	33.80	C-F	56.16	CDE	97.52	CD	116.33	CD	122.88	B-E	126.88	B-E	130.57	BCD
	KKU−SB052	24.66	G	47.56	E	98.85	CD	120.65	BCD	119.44	CDE	125.87	B-E	126.87	CDE
	SJ5	27.87	FG	51.79	DE	67.83	CD	111.6	DE	115.97	DE	117.21	E	118.60	E
Moderate	KKU−SB022	44.51	AB	71.98	AB	105.32	BC	123.22	BC	126.95	BCD	131.47	BCD	132.84	BCD
	SK1	37.92	B-E	66.54	ABC	101.62	CD	120.59	BCD	127.82	BC	129.62	BCD	130.88	BCD
	KKU−SB057	34.30	C-F	52.12	DE	82.33	E	102.12	E	112.28	E	116.98	E	119.27	E
	SJ2	33.29	C-G	54.89	CDE	97.12	CD	117.49	BCD	129.72	BC	136.02	AB	140.35	AB
	CM60	45.28	AB	66.85	ABC	69.29	CD	112.93	CDE	123.06	B-E	128.42	BCD	131.58	BCD
Susceptible	CM6	43.72	AB	77.71	A	119.41	AB	136.21	AB	141.75	A	143.52	A	144.79	A
	SK2	39.96	BCD	63.62	BCD	99.74	CD	117.54	BCD	129.26	BC	133.10	A-D	135.18	ABC
	LP	42.02	ABC	65.73	ABC	98.12	CD	114.25	CD	120.52	CDE	123.19	DE	123.98	DE
	RJM	32.31	D-G	58.77	CDE	97.98	CD	119.04	BCD	127.65	BC	131.73	BCD	134.61	A-D
	SJ1	29.31	EFG	52.04	DE	90.96	DE	114.69	CD	125.22	BCD	131.31	BCD	135.51	ABC
	Mean	36.62		61.49		99.71		118.12		129.03		129.03		131.19	
	CV (%)	17.04		14.19		8.73		7.11		5.90		5.90		5.94	
	F-test	**		**		**		**		**		**		**	
	LSD	8.89		12.42		12.39		11.96		10.83		10.83		11.10	

Table S1. Effects of different periods of flooding stress on imbibition curve in 15 soybean varieties (Cont.)

Groups	Varieties	Mass increment (%)					
		24 hours		36 hours		48 hours	
Tolerant	KKU–SB002	128.17	D-G	128.29	DE	129.98	CDE
	KKU–SB009	137.07	A-D	137.23	A-D	139.99	ABC
	KKU–SB023	132.61	B-F	133.23	BCD	133.52	BCD
	KKU–SB052	130.72	C-G	133.08	BCD	133.24	A-D
	SJ5	120.32	G	121.55	E	121.55	E
Moderate	KKU–SB022	136.00	A-D	133.39	A-D	139.75	ABC
	SK1	132.29	C-F	132.29	CDE	132.29	B-E
	KKU–SB057	125.10	EFG	128.39	DE	131.10	B-E
	SJ2	143.02	AB	143.67	AB	146.30	A
	CM60	132.03	CD	131.72	CDE	132.03	B-E
Susceptible	CM6	145.30	A	145.30	A	145.80	A
	SK2	135.82	A-E	135.99	A-D	135.99	A-D
	LP	132.98	FG	126.65	DE	126.65	DE
	RJM	137.19	A-D	137.19	A-D	137.19	A-D
	SJ1	139.94	ABC	142.18	ABC	142.18	AB
	Mean	139.94		134.21		135.30	
	CV (%)	5.64		5.69		5.86	
	F-test	**		**		**	
	LSD	10.71		10.88		11.29	

Table S2. Original soybean names of soybean germplasms used in this study. A total of 74 accessions were evaluated in the preliminary screening, from which 15 accessions were selected for the experiments reported in the manuscript.

No	Accession number in Khon Kaen University	Original varieties name	Country of origin	Sources
1	KKU–SB002	Athow	USA	NIAS
2	KKU–SB009	CHIENEUM KONG	Korea	NIAS
3	KKU–SB022	CHIENGMAI PALMETTO	Thailand	NIAS
4	KKU–SB023	BARITOU 3 A	Indonesia	NIAS
5	KKU–SB052	Tidar	Indonesia	NIAS
6	KKU–SB057	M 44	India	NIAS
7	KKU–SB062	Chiang Mai 60	Thailand	Thai DOA
8	KKU–SB066	SJ5	Thailand	Thai DOA
9	KKU–SB125	SJ2	Thailand	Thai DOA
10	KKU–SB126	Sukhothai1	Thailand	Thai DOA
11	KKU–SB127	Sukhothai2	Thailand	Thai DOA
12	KKU–SB129	Chiang Mai 6	Thailand	Thai DOA
13	KKU–SB131	Lopburi 84–1	Thailand	Thai DOA
14	KKU–SB132	Rajamagala1	Thailand	Thai DOA
15	KKU–SB145	SJ1	Thailand	Thai DOA

*Abbreviations: DOA is the Department of Agriculture
